# Independent Heath Facility Meets Cancer Care Ontario and Canadian Association of Gastroenterology Guidelines for Endoscopic Procedure Wait Times While Meeting Quality Indicators: A Retrospective Review

**DOI:** 10.1155/2018/4708270

**Published:** 2018-06-03

**Authors:** Fraser Kegel, Niv Sne, Timothy Rice, Eric Joy, Shayan Shahsavar, Celeste A. Collins, Maria Gagarine, Alexandra Allard-Coutu, Lisa Klotz, Angela Coates, Kamyar Kahnamoui, Marko Simunovic

**Affiliations:** ^1^Faculty of Health Sciences, McMaster University, Hamilton Health Sciences, Hamilton, Ontario, Canada; ^2^Faculty of Medicine, McGill University, Montreal, Quebec, Canada; ^3^Division of General Surgery and Trauma, McMaster University, Hamilton Health Sciences, Hamilton, Ontario, Canada; ^4^GHA Surgical Centre, Hamilton, Ontario, Canada; ^5^Michael G Degroote School of Medicine, McMaster University, Hamilton, Ontario, Canada; ^6^University of Ottawa, Canada

## Abstract

**Background:**

Canadian independent health facilities (IHFs) have been implemented to reduce hospital endoscopy volume and expedite endoscopic evaluations for patients suspected to have underlying colorectal cancer.

**Methods:**

We conducted a retrospective review of a prospective database at a large-volume urban IHF. The primary outcomes were wait times, and the secondary outcomes were colonoscopy quality indicators and complication rates.

**Results:**

Median wait times from referral to colonoscopy met the recommendations set out by the Canadian Association of Gastroenterology and Cancer Care Ontario for all indications: chronic abdominal pain: 43 days; new onset change in bowel habits: 36 days; bright red rectal bleeding: 42 days; documented iron-deficiency anemia: 43 days; fecal occult blood test positive: 38 days; cancer likely based on imaging or physical exam: 23 days; chronic diarrhea and chronic constipation: 42 days; and screening colonoscopies: 55 days. Secondary outcomes of quality indicators and complication rates all met or exceeded the CCO and CAG recommendations.

**Conclusions:**

This IHF met the recommended wait times for all indications for colonoscopy while maintaining high procedural quality and safety. IHFs are one solution to help meet the increasing demand for colonoscopy in Ontario.

## 1. Introduction

Colorectal cancer (CRC) is the second most common cancer in men, and the third most common cancer in women [1]. CRC is estimated to represent 1 in every 8 cancer cases and deaths due to cancer in Canada in 2016 and it is expected that 1 in 15 men and 1 in 16 women will develop CRC during his or her lifetime [1]. Since 2000, however, there has been a decrease in the incidence of CRC among the Canadian population, likely attributable to improved provincial screening programs [1]. Similarly, CRC mortality rates in the United States have dropped by approximately 25%, largely due to earlier detection of lesions and improved treatment [2]. Most colorectal cancers develop from adenomatous polyps, and these lesions are readily detectable using colonoscopy [2]. The Canadian Association of Gastroenterology (CAG) Wait Time Consensus Group recommends that patients with symptoms concerning for colorectal malignancy should have an expedited endoscopic examination [3]. It is recommended that patients with a high likelihood of CRC based on physical examination or abdominal imaging undergo consultation within two weeks and a definitive diagnostic workup within four weeks, and that patients who present with symptoms of bright red blood per rectum (BRBPR), documented iron-deficiency anemia, one or more positive fecal occult blood tests (FOBT) or fecal immunochemical test (FIT), chronic constipation or chronic diarrhea, new onset change in bowel habits, or chronic unexplained abdominal pain undergo endoscopic examination within 2 months [3, 4].

The CAG recommendations for acceptable wait times for consultation and endoscopic evaluation of patients with digestive symptoms and their subsequent survey to access of care suggest clinicians across Canada continually fail to meet the assigned times [4]. In order to expedite workup and referral patterns for patients with signs and/or symptoms suggestive of underlying colorectal cancer, Cancer Care Ontario (CCO) developed a new set of guidelines to achieve this. The CAG wait time guidelines are consistent with the CCO guidelines for the care of patients suspected to have colorectal cancer [3, 4]. The reported practice audits since the establishment of the CAG wait time guidelines have been shown to be consistently longer than the recommended wait times [5–7]. Over the past decade, there has been a trend toward endoscopy services being provided in community-based independent health facilities (IHFs) to aid in resource accessibility for patients.

Few studies have evaluated wait times or other quality markers among patients receiving endoscopic care at IHFs. We wished to evaluate CAG and CCO wait time recommendations and other quality marker recommendations in an urban IHF.

## 2. Materials and Methods

### 2.1. Study Setting

This study involved a retrospective review of a prospectively collected and maintained database of endoscopic procedures performed from 2014 to 2015 at a community IHF. Data collection was conducted by authors who did not perform or observe the endoscopic procedures. This IHF is a free-standing facility in an urban neighbourhood in Ontario and offers both gastroscopy and colonoscopy to patients. It is medically staffed by thirteen general surgeons, one gastroenterologist, and fifteen anesthetists. Over 95% of the clinicians are affiliated with a hospital appointment which is tied to an academic centre, and since its inception, this IHF has been open to both gastroenterologists and general surgeons. This IHF is accredited by the College of Physicians and Surgeons of Ontario (CPSO) and routine quality assurance evaluations are conducted on a biannual basis.

### 2.2. Study Population

**Figure 1:**
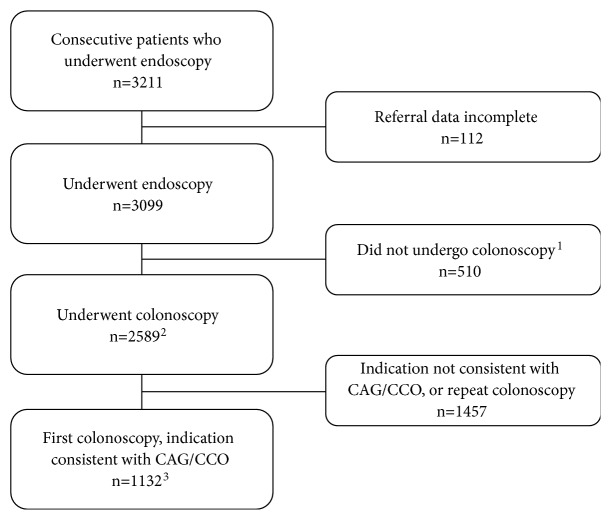
Patient selection. CAG: Canadian Association of Gastroenterologists, CCO: Cancer Care Ontario, ^1^patients underwent an endoscopic procedure other than a colonoscopy, ^2^patients included in secondary analysis (quality indicators and complication rates), and ^3^patients included in primary analysis (wait times).

Demographic information was collected from all consecutive patients from February 2014 to June 2015. Patients referred by a primary care physician for their first colonoscopy to investigate gastrointestinal symptoms, abnormal physical examination, or radiological findings suspicious of CRC as well as asymptomatic patients referred by a primary care physician for their first colonoscopy who met screening criteria for CRC or to investigate fecal occult blood test positivity were included in the analysis of primary outcomes, which in this study were wait times. Patients who were referred from a specialist physician, patients who have undergone previous colonoscopy, patients who did not undergo a colonoscopy, or patients with a medical record without referral information were excluded from the wait times analysis. The analysis of secondary outcomes included all patients with complete medical records who underwent colonoscopy. No patients were denied a consultation after referral. Demographic data including age, sex, endoscopist specialty, and result of bowel preparation were collected. The patient selection process is described in Figure 1.

### 2.3. Outcomes

Our primary outcome was wait times from referral to consultation, from consultation to procedure, and from referral to procedure for patients referred from a primary care physician undergoing a first-time endoscopy. For each patient, the referral method (either from a primary care physician or a specialist physician), referral date, consultation date, and procedure date were recorded.

Our secondary outcomes were quality indicators and complication rates. Bowel preparation was graded as good (no stool or small amounts of stool which can be easily cleared), fair (small amounts of semisolid stool that cannot be easily cleared), or poor (semisolid or solid debris that cannot be completely cleared). The most proximal anatomical structure intubated was recorded for each patient and if the cecum was unable to be intubated, reasons were recorded. Cecal intubation was confirmed by direct visualization of the appendiceal orifice or ileocecal valve or by ileal intubation. Digital photographs were obtained of the cecum for each successful cecal intubation. A random sample of 20% of the digital cecal images was reviewed by blinded authors to ensure the cecum was reached when it was reported to have been reached. One hundred percent of procedures whereby the most proximal point reached was recorded as the cecum were deemed correct according to the digital image audit. Polyp removal data was taken from colonoscopy reports and was confirmed from pathology reports. In adherence to the CPSO guidelines in Ontario for IHFs, any adverse events requiring patient transfer to an acute care hospital were recorded and are submitted regularly to the CPSO for review. All periprocedural complications are tracked. All patients received both verbal and written instructions to seek immediate medical attention if any untoward respiratory, cardiac, gastrointestinal, systemic, or other symptoms arose and to inform the IHF of such events. Time taken to reach the most proximal anatomic structure and total time for colonoscopy completion were recorded. Finally, the presence and location of any additional endoscopic findings were recorded.

### 2.4. Analysis

Detailed patient demographic and procedural information was collected from an electronic medical record system which requires encrypted passwords. The data was collected using an electronic data collection sheet which then compiled data into a spreadsheet for analysis. Statistical analysis was performed with SPSS Version 24 statistical software (IBM SPSS Statistics, IBM Corporation, Chicago, IL). The wait times are presented as the median days with 95% confidence intervals and are defined according to the previous surveys [6, 7]. Wait time to consultation is defined as the time from when the patient was first referred to the IHF until the time of consultation; wait time to procedure is defined as the time from when the patient first consulted the IHF health care provider until the time of completion of the colonoscopy; and, total wait time is defined as the time from when the patient was first referred to the IHF until the time of completion of the colonoscopy. Due to the nature of the confidential medical records at this IHF, patient names and medical record numbers were not obtained during the data collection process, and as such the McMaster University Research Ethics Board indicated that institutional review board approval was not required.

## 3. Results

### 3.1. Primary Outcomes: Wait Times

At this IHF, data was collected from 3211 consecutive patients during the study period. Of these, 1132 patients underwent a first-time colonoscopy after referral from a primary care physician for indications consistent with CCO and CAG and were included in the analysis of the primary outcomes (wait times) (Figure 1). Demographic data is available in Table 1. The wait times for all colonoscopy indications were shorter than the CAG and CCO recommendations and almost all colonoscopy indications were shorter at the IHF compared to the wait times reported in the Society of American Gastrointestinal and Endoscopic Surgeons (SAGES) 2008 and 2012 surveys (Table 2).

### 3.2. Secondary Outcomes: Quality Indicators and Complication Rates

There were 2589 consecutive patients who underwent either a first-time or repeat colonoscopy who were referred by either a primary care or specialist physician (Table 1). These patients were included in the analysis of secondary outcomes (quality indicators and complication rates). The mean age was 56.2 years (95% CI 55.7-56.6, range 19-84) and 49.8% were female. Over 98% of the colonoscopies were performed by a general surgeon. The quality of colonoscopy preparation was observed to be good for 74.4% patients, fair for 19.6%, poor for 5.8%, and not reported in 0.2%. Indications for colonoscopy are shown in Table 3. The proportion of patients who underwent colonoscopy for BRBPR at the IHF (18.5%) were higher compared to those reported in SAGES 2008 (9.2%) and SAGES 2012 (9.5%) surveys [6, 7]. As well, the proportion of patients who underwent screening colonoscopy at this IHF (34.2%) were higher compared to the SAGES 2008 (19.4%) and SAGES 2012 (21.0%) surveys [6, 7]. The remaining proportions of colonoscopy indications were comparable. Secondary outcomes of quality indicators all exceeded the CAG recommendations of all patients. The cecal intubation rate was 97%, the rate of inadequate bowel preparation was 6%, and there were no periprocedural complications (Table 4). In screening colonoscopies, the polypectomy and adenoma detection rates were 44% and 40%, respectively, for male patients, and 34% and 30% for female patients. The secondary outcomes for patients who underwent a first-time colonoscopy after referral from a primary care physician also exceeded the CAG recommendations. The cecal intubation rate was 98%, the rate of inadequate bowel preparation was 7%, and there were no periprocedural complications (Table 4). In this cohort, patients who underwent colonoscopies indicated for screening for CRC had polypectomy and adenoma detection rates of 36% and 25%, respectively, for male patients, and 34% and 19% for female patients.

## 4. Discussion

There has been an increase in demand for colonoscopy in Canada over the last number of years which is reflected by the greater number of colonoscopies that are being performed across Canada on an annual basis. The number of required colonoscopies may increase with the wide spread implementation of FIT and the increasing aging population [11]. As a result, patient wait times from evaluation of symptoms concerning for gastrointestinal malignancy to diagnostic endoscopy are lengthening. In addition, the wait times are longer than the recommended maximum wait times set forth by the CAG and CCO [12, 13]. In 2010, Sey et al. reported that 46% of patients diagnosed with colorectal malignancy (N = 106) in London, Ontario, were subject to wait times longer than the recommended standard of care [14]. Similarly, in a 2015 Canadian hospital-based practice of ten gastroenterologists, Janssen et al. reported that 58% of patients diagnosed with colorectal cancer (N = 246) experienced wait times longer than the recommended 60 days [13]. It has been suggested that the use of IHFs to provide patients with endoscopy is an option to decrease patient wait times by offsetting the workload of hospital-based endoscopic practices. In 2011, Ivers et al. reported shorter wait times for screening colonoscopies, but not for symptomatic patients, at nonhospital clinics compared to hospital settings [15]. This IHF has demonstrated that wait times for patients presenting with symptoms, abnormal test results, and physical examination findings suspicious for GI malignancy undergo diagnostic endoscopy well within the CCO recommendations. Similar findings were noted for patients who were asymptomatic but met the criteria for screening colonoscopy based on either family history, age, and/or positive FOBT results.

The evidence of the quality and safety of endoscopic procedures performed in nonhospital settings does not demonstrate consistency. In 2007, Bressler et al. found that the risk of postcolonoscopy colorectal cancer (PCCRC) rates was higher in office-based colonoscopy procedures compared to hospital-based procedures [16]. Shah et al. reported a higher odds ratio (3.57) for an incomplete procedure during colonoscopies performed in a private setting in 2007 [17]. In 2013, Chukmaitov et al. found a higher adjusted risk (1.27) of serious complications (colonic perforation or gastrointestinal bleeding requiring hospitalization) occurring after colonoscopy performed in ambulatory surgery centres compared with hospital outpatient departments [18]. Conversely, several studies have shown that colonoscopies performed in nonhospital settings are, in-fact, safe, of high quality, and performed within appropriate wait time guidelines. In an Ontario-based study in 2009, Bair et al. concluded that colonoscopies performed in IHFs by well-trained physicians were safe and of high quality [19]. Kozbial et al. demonstrated, in 2015, that endoscopists were able to provide high quality colonoscopies in both hospital and office-based settings [20].

In this study, the adenoma detection rate in all patients was found to be lower in women (30%) compared to men (40%). This has been shown in the literature [21] and since men have a higher prevalence of colorectal cancer compared to women, it is reasonable that men have a higher rate of adenoma detection.

During the study period at this IHF, nearly all colonoscopies were performed by general surgeons. This should not affect patient outcomes, as shown in a recent comparison between surgical and nonsurgical endoscopists; there was no significant difference in cecal intubation rate, adenoma detection rate, or polypectomy rate, and surgical endoscopists reported lower rates of hemorrhage [20].

It is important to ensure that the quality and safety of endoscopic procedures are not compromised when meeting the recommended wait times. This study demonstrates that this IHF meets the predetermined quality indicators as outlined by CCO and CAG.

The patients included in the analysis of the primary outcomes were a subset of the patients included in the analysis of the secondary outcomes (quality indicators and complication rates). Patients who underwent a repeat colonoscopy were removed from the wait times analysis to eliminate bias from patients undergoing scheduled procedures at yearly intervals, which would misrepresent the true wait times. In order to conduct the most thorough analysis of colonoscopy quality indicators and complication rates, all patients who underwent colonoscopy regardless of referral pattern were included in the analysis of secondary outcomes.

Although this review was obtained from a prospectively maintained database, it was retrospective in nature and, thus, lends itself to the biases associated with such studies. Additionally, this IHF does not track PCCRC rates; however, every endoscopist in Ontario is audited according to the same criteria, regardless of location of practice. The following data are measured and tracked for each endoscopist in Ontario: total colonoscopy volume per endoscopist, inadequate bowel preparation rate, outpatient polypectomy rate, outpatient cecal intubation rate, postpolypectomy bleeding rate, outpatient perforation rate, CRC detection rate, PCCRC rates, and adenoma detection rate [8]. This IHF utilizes a quality assurance program as mandated by the CPSO that ensures endoscopy quality is maintained while providing high volume care. The capture of patients who do not undergo colonoscopy after consultation remains a potential weakness of this study. This study is strengthened by the large population size and that those involved in data collection and analysis were blinded to the colonoscopy procedures.

Utilizing IHFs for endoscopic evaluation of patients will decrease in hospital endoscopy volume and decrease overall wait times. Moving forward, IHFs should continue to report colonoscopy indications, wait times, quality indicators, and complication rates in order to further evaluate the efficacy of IHFs. The CRC screening program in Ontario may utilize more FIT compared to FOBT because the former has been shown to increase quality adjusted life years at a lower cost compared to latter [22]. However, since FIT is more sensitive but less specific compared to FOBT [22], the volume of colonoscopy for patients aged 50-74 may increase.

Colorectal malignancy is a very common and serious malignancy. Although the cause of prolonged wait times for endoscopy in Ontario, and Canada, is multifactorial, one solution is the increased implementation and utilization of IHFs. This IHF reported providing endoscopy to patients well within the recommended wait time guidelines. Furthermore, the quality and safety of these endoscopic procedures are comparable to recommended set points. Finally, although these results are promising, we should be cautious about extrapolating this data to additional IHFs and encourage other facilities to report wait times, quality indicators, and complication rates.

## Figures and Tables

**Table 1:** Patient demographics.

Demographic	Patients included in primary outcomes (wait times) analysis	Patients included in secondary outcomes (quality indicators and complication rate) analysis
N	1132	2589
Age in years, mean (95% CI, Range)	54.8 (54.0-55.6, 19-84)	56.2 (55.7-56.6, 19-84)
Female, %	51.3	49.8

Endoscopist, %		
General surgeon	98.7	99.0
Gastroenterologist	1.3	1.0

Bowel preparation, %		
Good	73.6	74.4
Fair	19.5	19.6
Poor	6.6	5.8
Not reported	0.3	0.2

CI: confidence interval.

**Table 2:** Overall wait time indications for colonoscopy for this IHF, the SAGES 2008 survey, and the SAGES 2012 survey, and recommendations of Canadian Association of Gastroenterology and Cancer Care Ontario [3, 6, 7].

Indication	Study	Median days (95% CI)		
		Referral to consult	Consult to procedure	Referral to procedure
Chronic abdominal pain	IHF	26 (25-31)	18 (21-27)	43 (48-57)
	CAG/CCO			60
	SAGES 2012	102 (89-140)	67 (43-91)	153 (109-219)
	SAGES 2008	105 (91-119)	44 (28-72)	152 (104-198)

New onset change in bowel habits	IHF	16 (18-26)	18 (19-27)	36 (39-52)
	CAG/CCO			60
	SAGES 2012	84 (48-110)	49 (18-68)	103 (84-215)
	SAGES 2008	75 (63-90)	38 (19-68)	148 (98-210)

Bright red rectal bleeding	IHF	24 (24-29)	17 (20-24)	42 (46-52)
	CAG/CCO			60
	SAGES 2012	82 (52-104)	44 (32-64)	142 (92-181)
	SAGES 2008	58 (46-75)	54 (34-67)	136 (107-161)

Documented iron deficiency anemia	IHF	24 (23-29)	19 (20-26)	43 (45-53)
	CAG/CCO			60
	SAGES 2012	55 (40-73)	42 (29-58)	97 (62-160)
	SAGES 2008	56 (38-71)	35 (25-64)	90 (70-137)

Fecal occult blood test positive	IHF	16 (17-23)	15 (16-22)	38 (35-43)
	CAG/CCO			60
	SAGES 2012	56 (34-97)	50 (28-62)	105 (68-182)
	SAGES 2008	77 (61-92)	41 (30-82)	143 (122-219)

CRC likely based on imaging or physical exam	IHF	8 (9-34)	13 (9-23)	23 (19-55)
	CAG/CCO	14		28
	SAGES 2012	24 (8-59)	13 (1-42)	22 (6-182)
	SAGES 2008	72 (33-107)	36 (12-57)	82 (34-170)

Chronic constipation or chronic diarrhea	IHF	24 (23-31)	19 (20-28)	42 (45-56)
	CAG/CCO			60
	SAGES 2012	126 (103-141)	52 (30-64)	162 (116-221)
	SAGES 2008	119 (99-129)	57 (42-71)	186 (161-222)

Screening	IHF, for age	29 (32-36)	20 (25-29)	55 (58-64)
	IHF, for family history	30 (33-40)	20 (24-30)	54 (58-68)
	IHF, all screening	30 (33-37)	20 (25-29)	55 (59-64)
	CAG/CCO			180
	SAGES 2012	150 (130-174)	94 (70-128)	279 (239-321)
	SAGES 2008	127 (116-142)	72 (61-93)	201 (179-240)

IHF: this independent health facility, CAG: Canadian Association of Gastroenterology, CCO: Cancer Care Ontario, SAGES: Society of American Gastrointestinal and Endoscopic Surgeons, and CI: confidence interval.

**Table 3:** Indications for colonoscopy for this IHF, the Society of American Gastrointestinal and Endoscopic Surgeons 2008 and 2012 surveys [6, 7].

Indication	IHF 2017 N (%)	SAGES 2012 N (%)	SAGES 2008 N (%)
Chronic abdominal pain	238 (9.2)	181 (9.5)	205 (9.1)
New onset change in bowel habits	95 (3.7)	68 (3.6)	109 (4.8)
Bright red rectal bleeding	478 (18.5)	181 (9.5)	209 (9.2)
Documented iron deficiency anemia	194 (7.5)	102 (5.4)	132 (5.8)
Fecal occult blood test positive	112 (4.3)	65 (3.4)	79 (3.5)
Cancer likely based on imaging or physical exam	21 (0.8)	45 (2.4)	65 (2.9)
Chronic constipation or chronic diarrhea	160 (6.2)	160 (8.4)	229 (10.1)
Screening colonoscopy	903 (34.2)	398 (21.0)	438 (19.4)

**Table 4:** Quality indicators and complication rates as supported by a work group for the Cancer Care Ontario, and 2015 reported endoscopist performance statistics compared to this independent health facility (IHF) [8–10].

Quality indicators of colonoscopy	Patients included in primary analysis^1^	Patients included in secondary analysis^2^	Recommended	Provincial endoscopist performance, 2015
Cecal intubation rate, %	98	97	≥ 95	97 - 99
PR, screening, men %	36	45	-	34 - 50
PR, screening, women %	34	34	-	34 - 50
ADR, screening, men, %	25	40	≥ 25	-
ADR, screening, women, %	19	30	≥ 15	-
Inadequate bowel preparation, %	7	6	≤ 10	3 - 7
Withdrawal time^3^ (SD)	6 (5)	6 (5)	6-7	-
Complications				
Bleeding rate, %	0.0	0.0	≤ 0.1	0.1 - 0.5
Bowel perforation rate, %	0.0	0.0	≤ 0.1	0.1 - 0.8
Other rate, %	0.0	0.0	-	-

PR: polypectomy rate, ADR: adenoma detection rate, ^1^all patients undergoing first-time colonoscopy referred by a primary care physician, ^2^all consecutive patients undergoing colonoscopy, and ^3^mean withdrawal time in minutes.
